# Effect of supraglottic endotracheal tube inserted transorally on preventing hypoxemia in obese patients undergoing sedated bidirectional endoscopy: a prospective randomized controlled clinical trial

**DOI:** 10.1186/s12871-026-03608-2

**Published:** 2026-01-09

**Authors:** Guang-Qiu Zhu, Xiao-Xia Wang, Yu Wang, Hai-Tao Cong, Jing-Jun Jin

**Affiliations:** 1 Department of Anesthesiology, Taizhou Hospital of Zhejiang Province affiliated to Wenzhou Medical University, Linhai, Zhejiang Province 317000 China; 2 Department of Obstetrics, Taizhou Hospital of Zhejiang Province affiliated to Wenzhou Medical University, Linhai, Zhejiang Province 317000 China

**Keywords:** Hypoxia, Obesity, Bidirectional endoscopy, Sedation, Nasopharyngeal airway, Endotracheal tube

## Abstract

**Background:**

Prevention of hypoxemia in obese patients undergoing sedated gastroscopy is challenging, mainly due to upper airway obstruction. This study used a transorally inserted supraglottic endotracheal tube as an oropharyngeal airway to improve ventilation during sedated bidirectional endoscopy (esophagogastroduodenoscopy followed by colonoscopy) in patients with obesity.

**Methods:**

Patients with mild-to-moderate obesity (body mass index: 30–39.9 kg/m^2^) underwent bidirectional endoscopy during deep sedation and received supplemental oxygen via a modified oropharyngeal airway using an endotracheal tube (ETT-OA) (*n* = 97) or a nasopharyngeal airway (*n* = 96). An endotracheal tube was inserted through the side port of the endoscopic bite block into the supraglottic area under gastroscopic guidance. The primary outcomes were the incidence of hypoxemia and severe hypoxemia.

**Results:**

The minimum SpO_2_ was comparable between the two groups. The incidence of hypoxemia was significantly lower in the oropharyngeal airway group than in the nasopharyngeal airway group (4.1% vs. 14.6%, *P* = 0.012). Severe hypoxemia occurred in six patients in the nasopharyngeal airway group but not in the other group (*P* = 0.037). The distal end of the nasopharyngeal airway was not inserted into the supraglottic area in eight patients because the tube was short and could not be replaced with a larger-sized tube due to rhinostenosis. Epistaxis occurred in 12 (12.5%) patients in the nasopharyngeal airway group. Anesthesiologists and endoscopists were more satisfied with the oropharyngeal airway.

**Conclusions:**

An ETT-OA reduced the incidence of hypoxemia, especially severe hypoxemia, in patients with mild-to-moderate obesity during sedated bidirectional endoscopy compared with a nasopharyngeal airway.

**Trial registration:**

Chinese Clinical Trial Registry (ChiCTR2200064417), first registered on 07/10/2022.

## Background

Gastroscopy is usually performed under sedation to ease pain and ensure effective and timely completion [1, 2]. It often results in hypoxemia, a common and serious complication due to airway obstruction, respiratory depression, and the inevitable sharing of the upper airway space with the endoscope. Despite oxygenation via a standard nasal cannula (SNC), hypoxemia occurs in 13% of patients with normal body mass index (BMI) [3, 4] and 22% to 71% of obese patients [5–7]. A high BMI increases the risk of hypoxemia during anesthesia and sedation [8, 9]. With the high global prevalence of obesity and more patients with obesity undergoing gastroscopy [8], preventing hypoxemia in these patients is crucial.

Hypoxemia remains an issue, although many airway devices have been introduced to reduce the incidence of hypoxemia during gastroscopy in patients with obesity. Oxygenation with a high-flow nasal cannula (HFNC) can deliver an inspired oxygen fraction of up to 100%, producing a dead-space washout effect and a slightly positive end-expiratory pressure [10, 11]. However, HFNC demonstrates limited effectiveness in preventing hypoxemia in obese patients [12–14]. A recent meta-analysis of randomized controlled trials found that, compared to conventional oxygen therapy during gastrointestinal endoscopic procedures, HFNC did not significantly reduce the overall incidence of hypoxemia, though it may still benefit patients at moderate to high risk of desaturation [13]. This limitation is largely due to mouth opening during gastroscopy, which prevents the generation of sufficient positive airway pressure to overcome upper airway collapse. Various types of endoscopic and nasal masks can reduce the incidence of hypoxemia during gastroscopy. However, these are still challenging to use in patients with obesity due to upper airway obstruction [15, 16]. A nasal mask at a target continuous positive airway pressure of 10 cmH_2_O reduces the frequency and severity of hypoxemia in patients with obesity under deep sedation during colonoscopy [17]. However, 22% of patients with masks still require airway intervention.

A nasopharyngeal airway (NA) is usually used to maintain upper airway patency, reducing the incidence of hypoxemia [18, 19]. However, 2.8%–9.0% of patients who were not obese still required airway-opening maneuvers to correct hypoxemia during gastroscopic sedation using an NA [4, 20]. The Wei nasal jet tube (WNJT) is a new, unique NA that has helped reduce the overall incidence of hypoxemia in patients with obesity during gastroscopy from 38.5% to 8.1% [6]. However, the incidence of severe hypoxemia (2% vs. 7.7%) was comparable between the WNJT and nasal cannula, and 14.3% of patients with a WNJT required airway opening maneuvers [6].

In clinical practice, we face a challenge with NAs; their short length often fails to relieve upper airway obstruction in certain patients. Inserting larger NAs, which have increased length and outer diameter, is difficult or sometimes impossible due to rhinostenosis. When selecting an appropriate NA, the length is more important than the diameter [21, 22]. However, the length of the traditional NA is fixed for a given diameter. Moreover, the soft material of the NAs is prone to compression and deformation in narrow nasal passages, potentially causing new obstructions. To circumvent these issues and complications, we now use a transorally inserted endotracheal tube as a modified oropharyngeal airway (ETT-OA) device in the supraglottic area to secure upper airway patency during sedated gastroscopy.

This prospective, randomized study evaluated whether the ETT-OA prevents hypoxemia more effectively than an NA in obese patients during sedated bidirectional endoscopy (esophagogastroduodenoscopy followed by colonoscopy).

## Materials and methods

### Ethical considerations

The Ethics Committee at the Taizhou Hospital of Zhejiang Province approved this prospective randomized controlled clinical study (Approval no: K20220902), which was registered prior to patient enrollment in the Chinese Clinical Trial Registry (Registration no: ChiCTR2200064417, 07/10/2022). This trial was conducted between October 2022 and October 2023 at the Taizhou Hospital of Zhejiang Province. Written informed consent was obtained from all patients. All procedures complied with the ethical standards of the Institutional and National Research Committees and those enshrined in the Declaration of Helsinki (2013 amendment).

## Inclusion and exclusion criteria

The participants were Chinese patients with mild-to-moderate obesity scheduled for same-visit bidirectional endoscopy under deep sedation. Patients aged 18–65 years, with a body mass index (BMI) of 30.0–39.9 kg/m², having an American Society of Anesthesiologists (ASA) physical status of II, and being able to provide informed consent were included. Patients with the following conditions were excluded: a history of nasopharyngeal diseases (e.g., nasal polyps, epistaxis, and nasal deformities); coagulopathy; acute and chronic respiratory disorders (including chronic obstructive pulmonary disease and asthma); severe cardiovascular, cerebrovascular, hepatic, or renal diseases; pregnancy; pulse oxygen saturation (SpO_2_) < 90% without oxygen inhalation; and allergies to propofol, soybean, eggs, or albumin.

## Randomization and sample size estimation

An assistant not involved in this study used computer-generated randomization sequences and grouped them in a 1:1 ratio. The participants will be randomly assigned to receive oxygen through an NA or an ETT-OA. The sample size is determined online at http://powerandsamplesize.com/Calculators. We initially found that the incidence of hypoxemia was 14.5% in patients with obesity who were ventilated using an NA during a sedated bidirectional endoscopy. We assume that using an ETT-OA will reduce the incidence of hypoxemia from 14.5% to 3.0%. Given an α = 0.05 and a power of 80%, we estimate that 91 patients per group will be required for our study. Considering dropouts, the sample size is set at 200 (100 per group).

## Sedation protocol

All patients fasted for eight hours, after which their heart rates were monitored by electrocardiography. Noninvasive blood pressure and SpO_2_ were continuously measured. Topical oropharyngeal anesthesia was administered to all patients by gargling with 2% lidocaine. The nasal cavity of the NA group was sprayed with 2 mL of 2% lidocaine with epinephrine (1:100,000) for topical anesthesia. All patients were placed in the left lateral position with a head-up angle of 25°, fitted with an endoscopic bite block (Model:Ⅱ, Manufacturer: Taizhou Fuxing Medical Equipment Factory, Jiangsu, China), and pre-oxygenated via a face mask with an oxygen flow of 8 L/min for 3 min. The endoscopic bite block features a side port large enough to accommodate a standard 7.0-mm endotracheal tube. Sedation was then induced by a slow intravenous injection of 5 µg sufentanil (50 µg/mL; Yichang Humanwell Pharmaceutical, China) and 1.5 mg/kg propofol (10 mg/mL; Diprivan, AstraZeneca, Cambridge, UK), followed by additional boluses of 20‒40 mg titrated to achieve deep sedation. The sedation depth was evaluated using the Modified Observer’s Assessment of Alertness/Sedation (MOAA/S) score [23]. Deep sedation with a MOAA/S score 1 was initially implemented to decrease adverse responses to gastroscope tube insertion. The depth of sedation was continuously assessed at the bedside by the anesthesiologist using the MOAA/S scale and clinical signs. If MOAA/S scores were > 2 or the patient showed clinical signs of insufficient sedation during the procedure (such as body movement, gag reflex, or coughing), rescue sedation was administered with propofol titrated at 20‒40 mg as required.

The oxygen face mask was removed, and a Covidien Shiley nasopharynx airway (Covidien LLC, Mansfield, MA, USA) or an endotracheal tube (ID: 6.5–7.0 mm) was inserted as the ETT-OA for supplemental oxygen (5 L/min) when the sedation depth was adequate.

**Fig. 1:**
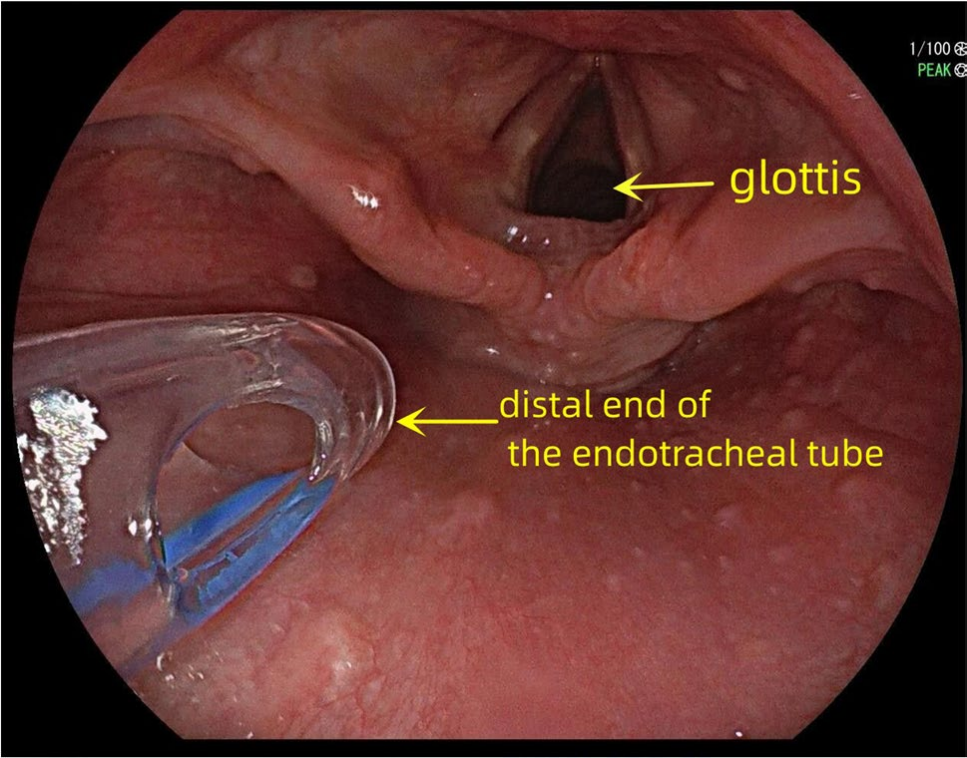
Position of the distal end of the ETT-OA in the supraglottic area

**Fig. 2:**
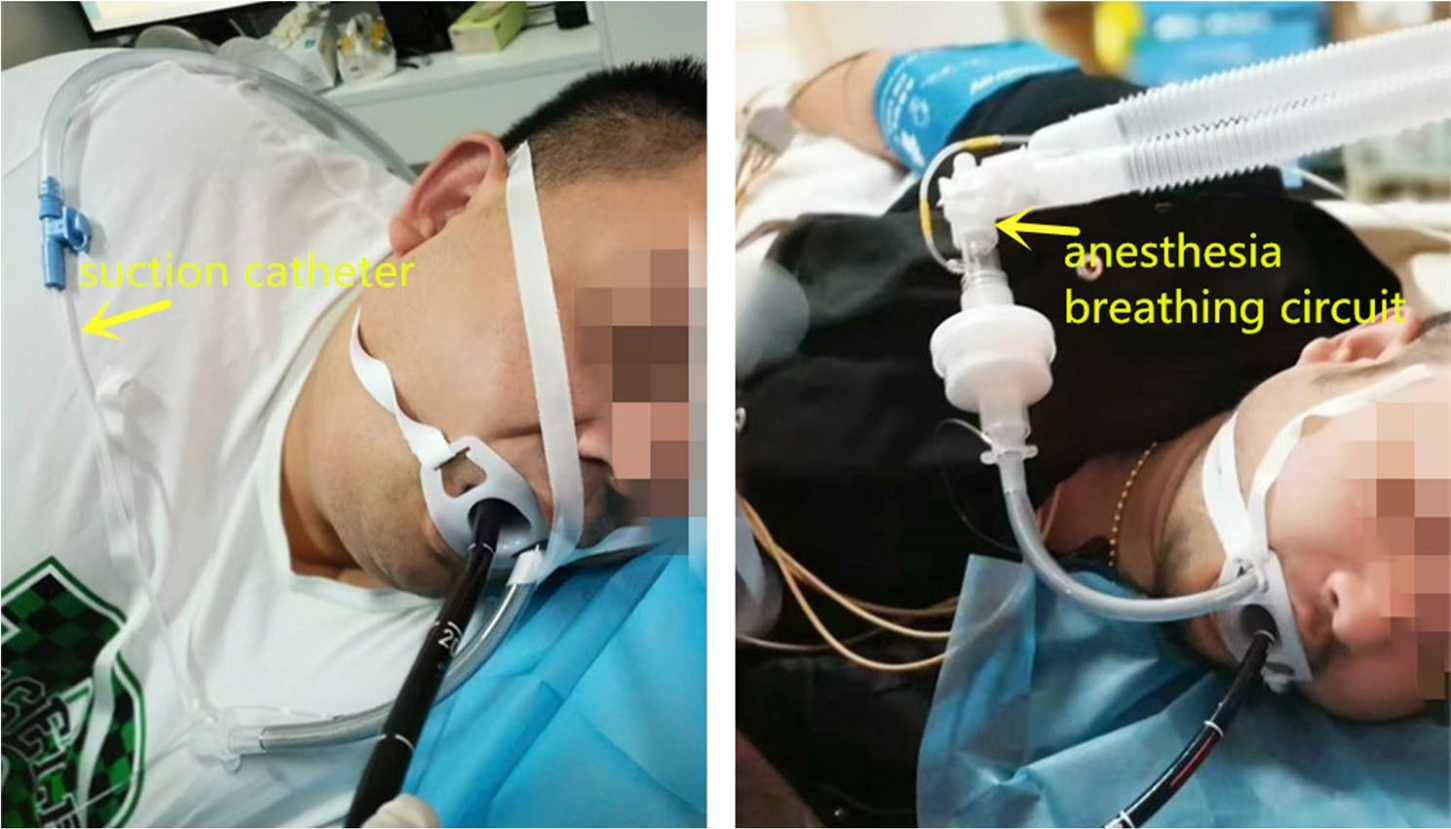
The ETT-OA connected to an anesthesia breathing circuit or a suction catheter for oxygen supply

A wire-reinforced endotracheal tube without stylet was used in the ETT-OA group. The tube was inserted through the side port of the endoscopic bite block and positioned in the supraglottic region between the epiglottis and the posterior pharyngeal wall under gastroscopic guidance (Fig. 1). The tip of the tube was lubricated with lidocaine cream and maintained at approximately 2 cm from the glottis to reduce mechanical irritation of the larynx. This precise placement is critical as it ensures patency of the airway, minimizes the risk of obstruction, and prevents potential laryngeal injury. The patient was placed in the “sniffing position,” a widely accepted maneuver for orotracheal intubation [24, 25], which helps pass the tube through the oropharynx in this procedure. The endotracheal tube can either be connected to an anesthesia breathing circuit or have a suction catheter inserted to provide oxygen, depending on whether an anesthesia machine is on standby (Fig. 2).

In the NA group, an appropriate NA size (ID: 6.5, 7.0, or 7.5 mm) was chosen based on the patient’s nostril size and the nare-to-tragus distance. The NA was lubricated with lidocaine cream before insertion. The more patent nostril (based on patient report) was attempted first. If insertion was difficult, the contralateral nostril was used. If still unsuccessful, a smaller-diameter tube was inserted. NA placement was considered a failure after four attempts. After the NA was secured to the skin, a lubricated suction catheter (10fr) was inserted into the distal end of the lumen as an oxygen supply pipe, or a smaller suction catheter (6fr) was inserted if the lumen was not sufficiently patent. The position of the distal end of the NA was examined using gastroscopy and adjusted appropriately.

The patients were transferred to the endoscopy recovery unit at the end of the procedure, and the artificial airways were removed after regaining consciousness. The endoscopists and anesthesiologists completed a satisfaction questionnaire after each procedure [26].

## Management of respiratory adverse events

Hypoxia-related adverse events were described as hypoxia (SpO_2_ < 90% during the procedure) and severe hypoxia (SpO_2_ < 75% or 75%≤SpO_2_ < 90% for 60 consecutive seconds or longer) [16]. Airway maneuvers such as chin lift and jaw thrust are executed in the event of hypoxia. The gastroscope was removed, and mask ventilation was initiated if hypoxia severity increased. Tracheal intubation was performed if the severity of the hypoxia remained unchanged.

Additional propofol (30–50 mg) was administered for laryngospasm [16]. In the event of regurgitation and aspiration, the gastroscope was withdrawn, oropharyngeal suction was immediately applied, and the patient was placed in the Trendelenburg position [16]. The aspirated gastric contents in the trachea were removed using tracheal intubation and fiberoptic bronchoscopy.

### Study outcomes

The primary outcomes were the incidences of hypoxemia and severe hypoxemia. The secondary outcomes included (1) minimum SpO_2_; (2) interventions required to manage hypoxemia, including airway opening maneuvers (such as head extension, chin lift, and jaw thrust), assisted ventilation, and emergency tracheal intubation; (3) other adverse events (such as epistaxis, laryngospasm, body movement, regurgitation, and aspiration); (4) endoscopy duration; (5) recovery time, calculated from the end of the procedure to the acquisition of a MOAA/S score of 5; (6) total propofol dosage; (7) lumen patency of the nasopharyngeal airway (assessed by inserting a 10fr suction catheter) and the distal position of the nasopharyngeal airway (assessed using gastroscopy); and (8) the endoscopist and anesthesiologist’s level of satisfaction with the procedure, rated as satisfactory, fair, or unsatisfactory, based on the occurrence of adverse events and the need for airway interventions.

## Statistical analyses

All data were statistically analyzed using SPSS 23.0 (IBM Corp., Armonk, NY, USA). Normally and non-normally distributed continuous variables are presented as mean ± standard deviation (SD) with 95% confidence intervals (CI) and medians with interquartile ranges (Q1–Q3). Categorical variables were expressed as numbers (%). The normality of continuous variables was assessed using Kolmogorov–Smirnov tests, and variance homogeneity was assessed using Levene tests. The characteristics and outcomes of the two groups were compared using the Student’s *t* test or Mann–Whitney U test for normally and non-normally distributed data, respectively. Proportions were compared between groups using the chi-square test. The satisfaction of anesthesiologists and endoscopists with the different protocols was analyzed using Wilcoxon rank-sum tests. Differences were considered statistically significant at two-tailed *P* < 0.05.

## Results

### Baseline demographics and characteristics

**Fig. 3:**
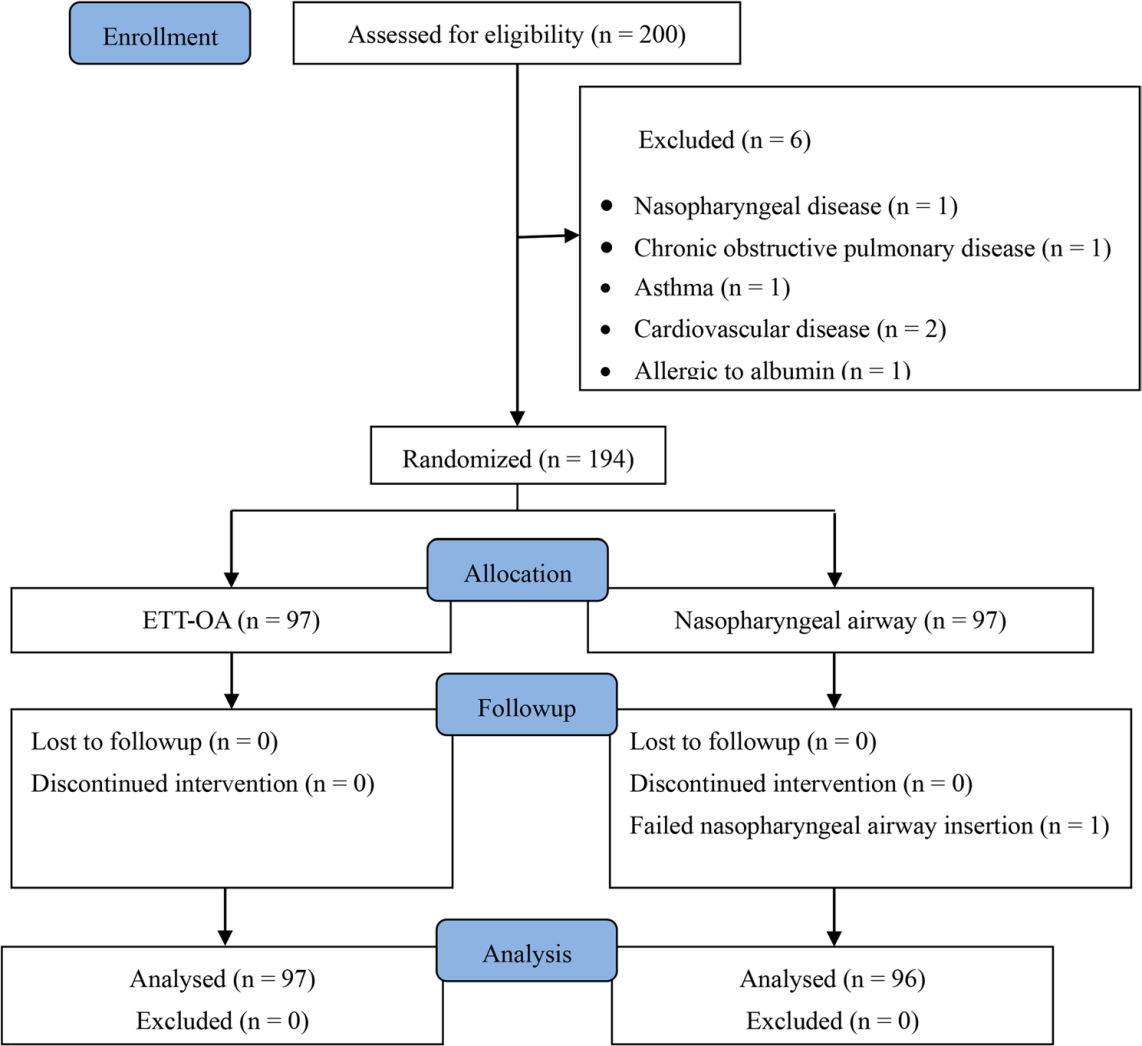
Flow diagram of the patients in this study

We initially enrolled 200 patients with mild-to-moderate obesity and then excluded six patients. Figure 3 presents a flow chart for study inclusion and exclusion. Thus, 194 patients were randomized into two groups (*n* = 97 each). However, one patient was excluded from the NA group because of rhinostenosis, which prevented NA insertion; therefore, data from 193 patients were analyzed. The demographic and baseline characteristics did not differ between the groups; endoscopy duration, total propofol dose, and recovery time were comparable (Table 1).

**Table 1:** Baseline demographics and characteristics of the procedures

Variable	ETT-OA (n = 97)	Nasopharyngeal (n = 96)	P
Age (y)	49 (41‒52)	47 (39‒51)	0.338
Sex (M/F)	66/31	53/43	0.067
BMI (kg/m^2^)	33.6 (30.1‒36.4)	33.8 (31.0‒37.0)	0.694
Baseline SpO_2_ (%)	96 (95‒97)	96 (95‒97)	0.649
Duration of endoscopy (min)	17.86 ± 3.95 (17.07–18.65)	16.91 ± 3.60 (16.19–17.63)	0.084
Total propofol dosage (mg)	350 ± 44 (341–359)	345 ± 36 (338–352)	0.379
Recovery time (min)	8.4 ± 2.4 (7.9–8.9)	8.7 ± 3.4 (8.0–9.4.0.4)	0.469

Data are presented as mean ± standard deviation (SD) with 95% confidence intervals (CI) and median with interquartile ranges (Q1–Q3) for continuous variables

*BMI* Body Mass Index, *SDs* Standard Deviations, *IQR* Interquartile Range, *SpO*_2_ Pulse Oxygen Saturation

### Oxygen desaturation outcomes and airway interventions

Table 2 details hypoxemia-related adverse events and interventions. The minimum SpO_2_ was comparable between the ETT-OA and NA groups (94% [93–96%] vs. 94% [90–96%], *P* = 0.114). However, the incidence of hypoxemia was significantly lower in the ETT-OA group than in the NA group (4 [4.1%] of 97 vs. 14 [14.6%] of 96, *P* = 0.012). Severe hypoxemia occurred in the NA group but not in the ETT-OA group (6 [6.3%] of 96 vs. 0 [0%] of 97, *P* = 0.037). None of the patients required endotracheal intubation.

**Table 2:** Oxygen desaturation outcomes, airway interventions, and other adverse events associated with ETT-OA and nasopharyngeal procedures

Assessments	ETT-OA (*n* = 97)	Nasopharyngeal (*n* = 96)	RR(95%CI)	*P*
Incidence of hypoxemia, *n* (%)	4 (4.1)	14 (14.6)	0.283(0.097–0.828)	0.012
Incidence of severe hypoxemia, *n* (%)	0 (0)	6 (6.3)	NA	0.037
Minimum SpO_2_, %	94 (93–96)	94 (90–96)		0.114
Airway opening maneuvers, *n* (%)	0 (0.0)	6 (6.3)	NA	0.037
Assisted ventilation, *n* (%)	0(0.0)	6(6.3)	NA	0.037
Body movement, *n* (%)	6(6.2)	8(8.3)	0.742(0.268–2.059)	0.565
Cough, *n* (%)	5(5.2)	4(4.2)	1.237(0.343–4.468)	1.000

Data are expressed as means with (SDs), medians with (IQRs) or number of patients

*SDs* Standard Deviations, *IQR* Interquartile Range, *RR* Relative Risk, *NA* Not Applicable

### Lumen patency and distal position of the NA

A 10-fr catheter could not be inserted in 2 (2.1%) of the 96 patients, possibly due to obstruction of the NA caused by rhinostenosis. Only a 6-fr suction catheter was inserted in these patients for oxygenation. Gastroscopy showed that the distal end of the NA was not inserted into the supraglottic area in 8 (8.3%) of 96 patients in the NA group because the tube was too short, and it was difficult to replace it with a larger size due to rhinostenosis. In the NA group, patients with severe hypoxemia had an abnormally positioned NA distal end and/or NA lumen obstruction.

### Other adverse events and procedure satisfaction

Gastroscopy identified epistaxis in 12 (12.5%) of 96 patients in the NA group but no apparent oropharyngeal mucosal injury or bleeding in the ETT-OA group. Other adverse events did not differ between the two groups (Table 2). None of the patients experienced laryngospasm, regurgitation, or aspiration. Anesthesiologists and endoscopists were significantly more satisfied with the ETT-OA than with the NA procedures (Table 3).

**Table 3:** Satisfaction of anesthesiologist and endoscopist with the protocol

	ETT-OA (*n* = 97)			Nasopharyngeal (*n* = 96)			*P*
	Good	Fair	Poor	Good	Fair	Poor	
Anesthesiologist, n(%)	92(94.8%)	5(5.2%)	0(0.0%)	82(85.4%)	10(10.4%)	4(4.2%)	0.025
Endoscopist, n (%)	86(88.7%)	11(11.3%)	0(0.0%)	79 (82.3%)	12(12.5%)	5(5.2%)	0.023

Data are expressed as number of patients

## Discussion

Hypoxemia is a common complication in patients with obesity who undergo sedated gastroscopy. Various airway devices, such as HFNC [12–14], endoscopic masks [15, 27], nasal masks [17], and NAs [6, 19], are effective in reducing the occurrence of hypoxemia. However, preventing hypoxemia using these airway devices for sedated gastroscopy in obese patients remains challenging. Compared to HFNC and WNJT used in obese patients during endoscopy, our ETT-OA technique achieved compelling results. The incidence of overall hypoxemia with ETT-OA (4.1%) was lower than the 8.1% reported with WNJT in a comparable obese population [6], and also compares favorably to outcomes from a large trial of HFNC in high-risk patients undergoing endoscopic procedures [28]. Notably, no severe hypoxemic events occurred in the ETT-OA group. Additionally, ETT-OA avoided nasal complications such as epistaxis, which is commonly associated with nasal devices. The LMA Gastro™ is a supraglottic airway device designed for use during gastroscopy. It has been shown to effectively reduce hypoxemia in high-risk procedures [29]. However, its insertion and retention typically require general anesthesia rather than deep sedation alone, as it is associated with high stress response and trauma [15]. Additionally, passing the endoscope through its gastric channel is sometimes difficult, which extends procedure time and increases operator fatigue, especially when using larger therapeutic scopes [30].

While previous studies have validated the use of an endotracheal tube as an OA across various contexts, ranging from animal models to clinical techniques such as the tube tip in pharynx (TTIP) for difficult airways [31–33], its application in digestive endoscopy has not yet been reported. Our study adapted TTIP for prophylactic use during sedated digestive endoscopy in obese patients, transforming it from a rescue maneuver into a preventive strategy against hypoxemia.

This randomized controlled trial assessed the efficacy and safety of a modified oropharyngeal airway using an endotracheal tube (ETT-OA) versus a conventional NA for supplemental oxygen delivery in adults with mild-to-moderate obesity undergoing sedated bidirectional endoscopy. We found that ETT-OA was more effective than NA in relieving upper airway obstruction. Therefore, ETT-OA reduces the incidence of hypoxemia, especially severe hypoxemia, greatly improving patient safety. The endotracheal tube is stiffer and longer than the NA tube, and the oral cavity is more spacious than the nasal cavity. Therefore, under gastroscopic guidance, the ETT-OA is easier to advance into the supraglottic area and is less susceptible to compression, deformation, or the development of new obstructions.

We also found that severe hypoxemia in the NA group was mainly due to a short NA that could not be inserted into the supraglottic area to relieve upper airway obstruction. The study participants from Southern China had relatively narrow nasal passages that restricted the selection of longer and larger-diameter NA. Consequently, in such patients with anatomically restricted nasal airways, ETT-OA significantly reduced intraprocedural hypoxemia (4.1% vs. 14.6%), translating into fewer airway rescue maneuvers (e.g., chin-lift, jaw-thrust) and no need for assisted ventilation, thereby improving procedural safety and workflow.

An ETT-OA does not cause nasal mucosal injury and is suitable for patients with a history of epistaxis, nasal polyps, and nasal deformities. However, patients with these conditions are unsuitable for NA insertion. In addition, endotracheal tubes, which are necessary for endoscopy centers, are less expensive than other airway devices. Therefore, the proposed method is both straightforward to implement and cost-effective.

Routine preoxygenation before anesthetic induction and tracheal intubation is a widely accepted strategy that aims to boost oxygen reservoirs, thereby delaying the onset of arterial hemoglobin desaturation during apnea [34]. Preoxygenation before esophagogastroduodenoscopy in patients with obesity is an evidence-based practice that can improve patient safety [35]. Preoxygenation in a 25° head-up position is more effective than in the supine position for obese patients, providing more oxygen storage in a larger lung volume and reducing the tendency for atelectasis and shunting [36–38]. Therefore, we applied preoxygenation in both groups using a 25° head-up position.

In this study, epistaxis in the NA group was 12.5%, which is consistent with the results of previous studies [39–42]. NA could not be inserted in one patient. In contrast, all insertions were completed without significant oropharyngeal mucosal injury in the ETT-OA group.

The satisfaction scores provided by anesthesiologists and endoscopists were mainly associated with the incidence of adverse events and the need for emergency airway management [6, 19]. The higher satisfaction scores with the ETT-OA may be attributed to reduced hypoxemia and fewer required airway maneuvers during gastroscopy.

This study had a few limitations. First, neither the medical personnel nor the research staff were blinded to group allocation. The lack of blinding could have led to performance and observer bias, a limitation we acknowledge in our study design. Second, the study population excluded patients with morbid obesity due to their higher risk of severe hypoxemia during sedated digestive endoscopy. Third, the patency of the nasopharyngeal airway was assessed only by suction catheter insertion rather than endoscopic verification.

In summary, this study demonstrated that the endotracheal tube-modified oropharyngeal airway (ETT-OA) outperformed conventional nasal airways in obese patients undergoing sedated digestive endoscopy. The ETT-OA was associated with reduced rates of hypoxemia, particularly severe episodes, and higher clinician satisfaction, establishing it as a safe and effective airway management option for this population.

## Data Availability

The datasets used and analyzed in the current study are available from the corresponding author in response to reasonable requests.

## Abbreviations

SNC: Standard nasal cannula
BMI: Body mass index
HFNC: High-flow nasal cannula
ETT: Endotracheal tube
NA: Nasopharyngeal airway
WNJT: Wei nasal jet tube
OA: Oropharyngeal airway
SpO2: Pulse oxygen saturation
MOAA/S: Modified Observer’s Assessment of Alertness/Sedation
TTIP: Tube tip in pharynx
